# A Thematic Analysis of Attitudes Toward Changes to Cervical Screening in Australia

**DOI:** 10.2196/12307

**Published:** 2019-04-11

**Authors:** Rachael H Dodd, Helena M Obermair, Kirsten J McCaffery

**Affiliations:** 1 School of Public Health Faculty of Medicine and Health The University of Sydney Sydney Australia

**Keywords:** screening, attitudes, cervical cancer, knowledge

## Abstract

**Background:**

In December 2017, the Australian National Cervical Screening Program (NCSP) was changed to encompass a 5-yearly human papillomavirus (HPV) primary test for women aged 25 to 74 years. Public concerns about changes to screening programs has been demonstrated in other countries previously.

**Objective:**

The aim of the study was to explore in depth women’s understanding of and concerns about the specific changes to the Australian NCSP implemented in December 2017.

**Methods:**

A Web-based petition (*Change.org*) opposing the changes received over 70,000 signatures and nearly 20,000 comments from February to March 2017. Of 19,633 comments, a random sample of 10% (2000/19,633) were analyzed using content analysis (reported elsewhere). Comments relating directly to the specific changes to the program were further analyzed using qualitative thematic analysis.

**Results:**

Around one-third (34.55%; 691/2000) of the total comments were related to concerns about specific changes to the program. The greatest concern was that screening intervals would be too long and that cancer may not be detected in time for successful treatment. Missing cancer in younger women (aged <25 years) was also an important concern, perceiving younger women to remain at significant risk. Notably, concern was rarely expressed about the new test (the HPV test).

**Conclusions:**

Gaps in knowledge and understanding about changes to the program and the rationale behind these have caused health concerns among women. Worry about the extended screening interval indicates little understanding of the slow progression of the HPV infection to cervical cancer or the high rates of regression. Identification of these knowledge gaps can inform both deintensification of other cancer screening programs and practitioners, so that they are able to address these concerns with their patients.

## Introduction

### Background

Cervical cancer is mostly attributed to the human papillomavirus (HPV), which is a virus transmitted through sexual contact [1]. From the time cervical screening was introduced in Australia in 1991, the number of women aged 20 to 69 years diagnosed with cervical cancer has fallen from 17 per 100,000 women to 9 per 100,000 women, and mortality rates have halved from 4 per 100,000 to 2 per 100,000 [2]. Incidence and mortality rates of cervical cancer in Australia and New Zealand are comparable with Western Europe and North America [3]. Testing for HPV has been utilized in cervical screening programs for triage and test of cure for women with cervical abnormalities (eg, United Kingdom), but many countries are now moving toward HPV screening as the primary test in cervical screening.

**Table 1:** The changes implemented to the Australian National Cervical Screening Program (NCSP) on December 1, 2017.

Change	New program (2017 to present)	Old program (1991 to 2017)
Test technology	The Cervical Screening Test takes cells from the cervix to test for human papillomavirus infection	The Pap test took cells from the cervix and examined these cells for physical changes
Interval	The Cervical Screening Test is every 5 years	A Pap test every 2 years
Age	Women will be invited for a Cervical Screening Test from the age of 25 years	Cervical screening began at 18 years of age
	Women will have their last Cervical Screening Test (*exit test*) between 70 and 74 years of age	Cervical screening ended at 69 years of age

Overdiagnosis and overtreatment are increasingly recognized as potential harms of screening, resulting in a need for screening programs to be reformed to ensure screening only occurs when benefits of early detection outweigh harms [4]. A renewed, deintensified National Cervical Screening Program (NCSP) was introduced in Australia in December 2017, which included a number of specific changes to the program (Table 1) [5]. The renewal based new recommendations on evidence of potential harms with the cytology (Pap test) program, in addition to data demonstrating success of the HPV vaccination and the development of new screening technology, which is more sensitive [6-8].

The deintensification of the NCSP has the potential to reduce overdiagnosis and overtreatment of cervical abnormalities and the additional harms associated with this. This is particularly relevant for women aged under 25 years, where incidence and mortality of cervical cancer is extremely low [2]; however, the transient nature of HPV in this age group results in women receiving potentially unnecessary and harmful treatment under the recommendations of the original program.

Although the changes were announced by the NCSP in April 2014, because there was no accompanying publicity, they went largely unnoticed by the public until February 2017 when a Web-based petition opposed to the changes was widely disseminated [9]. Similar hesitancy to changes in recommendations and deintensification of screening has been observed previously in the United States when the age of breast screening was increased from 40 to 50 years and the annual cervical screening interval was lengthened to every 3 or 5 years depending on the woman’s age [10,11]. Public consultations on the review of evidence toward the age of first screening and frequency of screening conducted by the UK National Screening Committee in 2012 have also demonstrated examples of such public concern [12]. Concern has also been expressed previously in Australia and Canada over delaying the age of screening [13,14] and changing the primary test to HPV testing [15,16]. The deintensification of screening programs is continually met with concern and opposition from the public, which can result in the recommendations being retracted [17,18].

### Objective

In our first study, we conducted a content analysis to identify and quantify the main themes and areas of concerns in women regarding the changes [19]. In this study, we explore in depth, women’s understanding and concerns about the specific changes and elements of the deintensified program. This will provide insight into the main concerns that need to be addressed as these changes are implemented and identify concerns that may be pre-empted for deintensifying other screening programs in the future in order to improve public communication strategies in screening.

## Methods

### Dataset

Comments posted on the *Change.org* petition, *Stop May 1st Changes to Pap Smears—Save Women’s Lives* (Multimedia Appendix 1), between February 16, 2017, and March 19, 2017, inclusively provided the dataset for this study [9]. Further information on the dataset and procedure is given in our previous publication [19]. Information given by each commenter included their name, state, city, and postcode. Of 2000 comments coded, over one-third (34.55%; 691/2000) reflected concerns about the specific changes to the cervical screening program recommendations. These comments represent the dataset on which the qualitative analysis was performed. This study was reviewed and approved by the University of Sydney Human Research Ethics Committee (project number 2017/300). Participant consent was not required as they had consented to their comments being freely available when they commented at *Change.org*.

### Analysis

A description of the content analysis from our first study is given in our previous publication [19]. The 2000 randomly selected comments were organized and coded in Microsoft Excel. Inter-rater reliability (Cohen kappa) between 2 coders (HO and RD) of the content analysis was 0.95, showing *nearly perfect* agreement [19]. Of 19 codes, 5 codes were related to specific changes to the screening program: opposition to the extended screening interval, concern about the increased age of the first invitation to screen, concern about missing cancer cases in older women, expressions of support for the current program, and disagreement with the HPV test itself. Comments relating directly to these 5 codes representing the specific changes to the cervical screening program were organized into worksheets in Microsoft Excel and then analyzed using qualitative thematic analysis [20]. This flexible approach gives theoretical freedom to analysis, enabling a rich and detailed account of the data. All the comments coded in each individual theme that related to the specific changes to the screening program were analyzed thematically. This analysis enabled the comments to be reviewed and defined in depth for further insight into the concerns expressed by commenters. Both coders of the data are women of screening-eligible age and acknowledge their own theoretical positions and values from a public health (postdoctoral researcher) and medicine influence (medical doctor).

## Results

### Overview

Among the 691 comments expressing concern about specific changes to the program, there was overwhelming support for the current cytology (ie, the existing program at the time of the petition) cervical screening program. Concerns about the renewed HPV primary screening program included (1) worry about the increased screening interval (from 2- to 5-yearly interval); (2) opposition to an increased age of the first invitation to screen to the age of 25 years; (3) disagreement with the change in test technology; and (4) worry about missing cases of cervical cancer in older women because of the introduction of the exit test.

### Support for the Current Cytology (Pap Test) Program

#### Keeping a Successful Program

Commenters viewed the current (cytology) cervical screening program as successful and therefore could not understand the reasons for changing a program that they know has been shown to be effective and save lives:

Vital to keep this system. It saves so many lives in Australia.

Some comments referred to the idea that the program was changing as a cost-saving measure, at the cost of saving lives:

The current system works very well, don't try and “fix” something that’s not broken to save money instead of saving lives.

#### Same Access to Screening for Future Generations

Commenters also mentioned a desire for future generations to have the same access to cervical screening that they have experienced. Commenters displayed no awareness of the concept of overdiagnosis and overtreatment, with the consistent belief that more screening saves lives and that it is always best to detect changes early. Commenters’ general understanding was that more screening equates to more lives saved:

I think it important that the current system remains as it is working. I have two daughters and would hope that the process was the same for them as it has been for me. More screening=early detection=lives saved.

### Opposition to an Increased Screening Interval

#### Prefer More Frequent Screening

This was the most concerning change for women (334/2000; 16.70%), with the most comments indicating concern that the 5-yearly interval between tests was too long compared with the 2-yearly interval (Table 2). There was a general preference expressed for annual or biannual screening, which was in some cases related to women’s own perception of increased risk owing to the experience of being diagnosed with abnormal cells:

I have a Pap smear every 2 years. I've needed to have abnormal cells removed. I changed to annual Pap smears for monitoring - nothing in 1 year to high risk in the next. This has happened to more women I know. It’s extremely common. This change to 5 years makes no sense.

#### Perceived Risk

Commenters also believed that this change would be putting more lives at risk, with the common concern that if a woman developed abnormalities within the 5-yearly interval, then these would already be cancer, suggesting women see having an abnormal Pap smear as having a *near-miss* with cancer:

This is a step backwards...How far could a cancer progress in the five years between testing. This is so ridiculous, just leave things that are working well alone.

These comments reflect a lack of understanding that cervical cancer develops slowly over a long period of time. It also illustrates confusion between precancerous cells and cancer. Commenters expressed the opinion that cervical cancer is a fast-progressing cancer and that with the introduction of a 5-yearly screening interval, this would leave many women at risk:

5 years is far too long for something as quick progressing as cancer, and given that young people (well under 25) are sexually active, they have a right to the protection that Pap smears offer just like everyone else.

#### Adherence to Recommendations

There was also suggestion that some may not follow the recommendations and that inequalities would arise owing to *only the rich* being able to pay to continue to have more frequent tests:

This is such an important test for early detection. The rich are fine for paying tests in between but why should it be at the detriment of middle and low income earners. It shouldn’t be the rich get treatment and those less fortunate die as they can't afford necessary tests when the government decides to increase the time between testing.

### Worry About Missing Younger Women

#### Significant Risk to Young Women

Another concern expressed was the change to the age in which women would be invited, with the starting age increasing from 18 to 25 years. Commenters expressed that they believed this change would lead to more deaths in young women and that women in this age group remain at significant risk of cervical cancer:

I don’t have much to say except this change is ridiculous. Chances are it will be responsible for the deaths of many young women.

**Table 2:** Concerns relating to the specific changes in recommendations.

Concerns^a^ and coded most with...		Example comments
**Want to keep current cytology (Pap Test) system**		
	Screening interval	There should be no change. Screening should be every two years.
	Women’s health	Pap smear testing is a vital health care service—so please leave it alone!! Women’s lives depend on it!!
	Prevention or early detection	I’m signing because early detection saves lives, why change something that has helped detect cervical cancer early.
**Worry about screening interval**		
	Personal experience	I have had abnormal Pap smear result which changed 2 levels in 9 months. Leaving it for 3 years would have meant death.
	Worry about missing young women	It should be decreased to once a year not increased to once every 5 years. The age should be decreased to 16 not increased to 25!!
	Women’s health	Women need their screening. 5 years is too long between screening. How many women have to end up with cancer before anything is done. Think about the women in your family.
	Prevention or early detection	I don’t agree with extending the time between tests. It should stay at every 2 years and that’s it. This will hopefully lead to early detection. A test 5 years apart…I can’t see how that can lead to early detection.
**Worry about missing young women**		
	Screening interval	I want Pap smears to be available to everyone from 18 years old every two years.
	Personal experience	A Pap smear detected pre-cancerous cells in my cervix when I was 20 years old. A delay of years could have compromised my survival.
	Worry about missing older women	Any form of cancer does not discriminate against age young or old can still get it & if a Pap smear saves 1 life that means it’s very worthwhile for all women of all ages.
**Disagreement with HPV^b^ test**		
	Screening interval	5 years is too long between tests for 'early' detection and limiting the test to only screen for HPV induced cancers will put a greater number of lives at risk.
	Women’s health	This is not fair to women all over Australia they should test for everything when giving us Pap tests, because otherwise they are putting us in danger and it’s not right.
	Worry about missing young women	When I start to get Pap smears, I want to trust that I’m being tested for ANY abnormalities, not just the 80% and I want to be able to start now, not in 7 years when I may already have abnormalities or cancer that could have been prevented and detected.
**Worry about missing older women**		
	Worry about missing young women	Screening should start as soon as girls are sexually active and certainly not finish at 70-75.

^a^34.55% of total sample.

^b^HPV: human papillomavirus.

Commenters believed if the age was to be changed, it should in fact be decreased because of this age group becoming more sexually active and that screening should start as soon as women become sexually active:

The 2 year Pap smear test should not change to 5 yearly, it’s putting women’s lives at risk. I think it should start early for younger women, especially if they are sexually active.

#### Personal Experience

Commenters had many examples of themselves, or someone else they knew having been diagnosed with cervical abnormalities under the age of 25 years, believing that had they or the woman they knew not been treated, cervical cancer would be inevitable. This reflects a gap in knowledge of the difference between cervical abnormalities and cervical cancer, with no awareness that cervical abnormalities can regress, particularly in younger women, often without requiring treatment:

A friend at age 19 during a regular Pap smear discovered cancerous cells—if she was meant to wait ’til 25 for her first one she would be dead.

Women also gave personal experiences as reasons for why the age of invitation should not be increased:

A Pap smear detected pre-cancerous cells in my cervix when I was 20 years old. A delay of years could have compromised my survival.

#### Comparison to Other Countries

A comparison was made to the age change to cervical screening in the United Kingdom, with the perception that many young women had died in the United Kingdom because the age of invitation was increased:

As for the age raising, this happened in the UK and there has been more and more young girls losing their battle because Paps are not even on their radar.

#### Symptomatic Screening

These comments reflected the notion that cervical cancer is always prevented through screening, with no commenters being seemingly aware that women still have the option of presenting to their doctors with symptoms, should these occur before women were invited for screening at the age of 25 years. As with the previous program, any woman presenting with symptoms can be screened outside of the screening program more frequently. These comments also reflect a gap in understanding that the vast majority of cervical abnormalities can regress without treatment.

### Disagreement With the Introduction of Human Papillomavirus Testing

#### Misunderstanding the Pap Test

The change of the test itself, from a cytology-based test (Pap smear) to an HPV test (cervical screening test), was rarely commented on. However, among those who expressed concern, worry related to a desire to monitor *all abnormalities* and not just HPV. This was coupled with a belief that the Pap test currently tests for several types of infection:

When I start to get Pap smears, I want to trust that I’m being tested for ANY abnormalities, not just the 80% and I want to be able to start now, not in 7 years when I may already have abnormalities or cancer that could have been prevented and detected.

Commenters were strong advocates for the Pap smear, believing that it detects all abnormalities compared with HPV test that was viewed as less thorough and not as advanced:

So far testing for HPV isn't advanced enough. And doesn't cover all cancers...I ask you to do what’s right and protect your women and keep the Pap smear testing unchanged.

Commenters continued to talk about screening in the context of Pap smears rather than HPV test:

Pap smears need to stay at two years…how dare a male run government make these decisions…it has been proven that age does not matter in these circumstances...

Commenters did not understand that HPV testing technology is a newer, more sensitive, and potentially sophisticated test than the Pap smear. They also believed that Pap smears detected other cancers as well as cervical cancer, when HPV testing will actually improve prevention of adenocarcinomas compared with the Pap smear:

It would be medically ignorant to make the changes you have suggested...Not all cervical cancer is caused by HPV and there are many types of cancers caught by the Pap smear testing.

#### Human Papillomavirus Vaccination

The HPV vaccine was also mentioned, with some recognition that the vaccine was already making a difference, but also with some understanding shown that the vaccine does not protect against all HPV types:

My understanding is that the HPV vaccination is only against 1 HPV & there are around 100 different HPVs.

There was recognition that a large proportion of the population (namely those older than the cohort offered the HPV vaccination) have not received the vaccine and commenters expressed the belief that the recommendations should be different for those who have not received the HPV vaccination:

I also understand MOST girls have now had the vaccination, perhaps those people who haven't, or don't know should at very least get a Pap early.

### Worry About Missing Older Women

This theme mainly reflected comments from women that *all women of all ages are at risk* and “age is no barrier.” This was the least coded concern from commenters.

Screening should start as soon as girls are sexually active and certainly not finish at 70-75.

## Discussion

### Principal Findings

This study presents an analysis of comments made to a Web-based petition opposing the changes to the Australian NCSP implemented in December 2017. This study focused on gaining an in-depth insight into comments opposed to the specific changes to the screening program, namely the extended screening interval, increased age of first screening, and the screening test itself. The greatest concern about the changes was reflected in comments opposing the extended interval between screening tests. Another important concern was the worry about missing cancer in young women owing to the later age of first screening, but the number of commenters showing concern about the new test (primary HPV testing) was minimal.

### Strengths and Limitations

The study benefits from rich data generated from a large-scale petition, with a sample of almost 20,000 comments. Although the commenters responding to the petition could be described as the *vocal minority*, this was the second largest petition in 2016 and 2017 on *Change.org* in Australia. Despite this, the vocal minority can result in change and negative press can be very powerful, such as in the United States where screening recommendations were retracted as a consequence [17,18]. Additionally, no demographic data were available for the commenters, so we cannot draw conclusions on the representativeness of the sample or give any detail about the commenters. Although the analysis of qualitative data is viewed as subjective, measures were taken to recognize sources of bias in the analysis by 2 authors coding the data, and comments from participants have been included in the results to support the interpretive findings.

### Comparison With Previous Work

The findings from this study build upon those from our previous content analysis [19] by adding a greater depth of analysis and providing more detail into women’s concerns about the specific changes to the NCSP. Although our previous study descriptively provides an overview of the opposition to the specific changes to the NCSP, this study discusses these further and reveals important concrete concerns.

Our findings support some prospective work conducted with a small cohort (n=149) of young Australian women (aged 16 to 28 years). This showed that although almost 79% were willing to screen with primary HPV testing, 65% were concerned about delaying cervical screening until the age of 25 years and 66% were unwilling to undertake screening with HPV testing from the age of 25 years, at 5-yearly intervals [13]. Extending the interval between cervical screens has also previously been found to be a concern for women in other countries [10,11,15] and was replicated in this study. Despite hesitancy from practitioners in Australia about the changes to the cervical screening program, encouragingly, if the changes were said to be recommended by the national guidelines, 60% have shown willingness to perform 5-yearly HPV testing from the age of 25 years [21]. The importance of practitioner support for a revised screening program is demonstrated by a US example, where despite a change in recommendations for cervical screening, health care providers still offer an annual Pap test [22,23].

Importantly, a number of misconceptions and gaps in knowledge about the progression of cervical cancer were apparent in the comments about the extended screening interval. Commenters expressed the belief that within the 5-yearly time frame between screening tests, it was likely that any cervical abnormalities could develop into cervical cancer, displaying a fundamental misunderstanding about both the natural history and progression of cervical cancer. This also demonstrates a failure to distinguish between precancerous abnormalities and cancer and no understanding about the high rates of regression of HPV and cervical abnormalities. Women therefore need to be educated about these issues, and primary practitioners are ideally placed to do this. Public awareness campaigns through social media may also be effective approaches given the increasing use of social media across the screening-eligible age. There were further gaps in knowledge about HPV testing technology, notably its sensitivity and its negative predictive value compared with the Pap smear, which is the rationale behind extending the screening interval. Previous research has shown that women with a better understanding of the rationale behind screening tests are more accepting of an extended screening interval [10,15]. In a sample of Canadian women, having a positive attitude toward the value of HPV testing was a significant predictor of willingness to participate in different screening regimens (HPV test, increased interval, and increased age of first screen) [14].

Missing younger women with cervical cancer owing to an increased age of the first screening invitation was a major concern. The common belief expressed by commenters in the petition was that women younger than 25 years of age were at increased risk of cervical cancer if they were no longer going to be screened. Not unsurprisingly, commenters showed no awareness of the concept of overdiagnosis and overtreatment in these younger women and did not have accurate knowledge about the low incidence of cervical cancer in younger women and declining rates of high-grade abnormalities. There has been little public information on these topics, and these views may also be a consequence of the high rate of attention given to younger women diagnosed or those who have died from cervical cancer in the media, for example, *Jade Goody*. Understanding the concept of overdiagnosis and overtreatment is fundamental to understanding some of the reasons behind the deintensification of this and future screening programs.

The consequences of overtreatment should be communicated to women so that they may understand more about the rationale behind increasing the starting age of screening. Previous research has shown that women who believe the extended interval is changing owing to scientific evidence rather than being driven by cost are more likely to accept the change [11]. Although some commenters demonstrated a misunderstanding of the difference between the previous and renewed screening program by expressing concern about missing cases of cervical abnormalities in older women, these concerns were not commonplace. This misconception is possibly because of the use of the *exit test* terminology, which sounds more final than previously, where women would simply not be tested after the age of 69 years. Women in Australia will now receive a screen at an older age than previously included in the screening program, which will assess those women at low risk and invite them to exit the screening program.

Women often do not remember being informed or are not aware of any changes in recommendations that occur to screening programs [24,25]. Very few commenters disagreed with the introduction of the HPV test and continued to refer to the Pap smear, which may reflect a lack of awareness about the change in test or a lack of understanding about the purpose of the test. Commenters also expressed a belief that the Pap smear tests for multiple infections and multiple cancers and did not seem to understand the purpose of the Pap smear as screening asymptomatic women [10], perhaps confusing the combination of the Pap smear with their Well Women’s Checks where other infections such as chlamydia are tested for but by using a different sample.

Health professionals have indicated their worry about the extended screening interval owing to women not attending regular health checks [22]. Previous research has indicated that between 56% and 75% of women would still attend regular Well Women’s Checks if the Pap smear was no longer at the same interval [13,24,25]. The hesitancy of these health professionals needs to be addressed as this could undermine patient education efforts if they continue to screen more frequently regardless of guidelines, such as in the United States where clinical practice has been slow to change [10,22,23] and many women are unaware about changes to the recommendations. A total of 60% of health professionals in Australia and New Zealand reported being willing to screen by the new guidelines, but stated they would be likely to screen women who are unvaccinated, are sexually active, or have a past history of cervical abnormalities, more often [21].

**Table 3:** Recommendations for health care practitioners to address with patients concerned about deintensification of screening programs

Change in screening program	Recommended information
Changing screening intervals	Some cancers can be very slow growing, taking between 5 and 10 years before growing to a point of causing a problem for a person’s health and so in some cases, might not cause any problems in a person’s lifetime. For example, human papillomavirus (HPV) is the main cause of cervical cancer, which is a very common infection where most sexually active people will pick up HPV at some point in their lives. In cervical cancer, only a small number of people who get HPV go on to develop abnormal cells and an even smaller number go on to develop cancer. Persistent infection with a cancer-causing type of HPV can cause abnormal cell changes that may lead to cervical cancer. However, this usually takes a long time, often more than 10 years. As tests that we use for cancer screening are now more accurate and sensitive, we can trust the results from these for a longer period of time. This means that if you are found to be at low risk, you do not need to be tested as frequently and can be more confident in the test results.
Reducing age range for screening	Cancer does not affect every age group the same. We now have extensive data about the number of cases of different cancers across the population and so we know which age groups are most at risk and would benefit most from screening. In some cancers, there can be more harm than benefit to screening younger age groups, as some abnormalities may be detected which would otherwise go away by themselves, or not cause harm in that person’s lifetime, but may lead to unnecessary treatment.
Changing screening technology	Owing to advancing technology, new tests are being developed which are more accurate and sensitive than previous tests. Some tests, such as the new cervical screening test, are also detecting changes at an earlier stage than the previous tests and will pick up any abnormal changes a stage earlier. The new cervical screening test is detecting HPV types which have the potential to cause cancer and the persistence of these HPV types, therefore detecting the virus that causes most cervical cancers.

Previous research has shown women are willing to be screened using the HPV test [13,16,24], particularly those who place more value on the national guideline recommendations [13], but that many do not understand what HPV testing is [26]. In response to the changing cervical screening program in Ireland, lacking knowledge about the test made it impossible for women to try to understand the reasons for any changes and make informed decisions about HPV testing [26]. It is conceivable that knowledge about how common HPV is, or an understanding that there is no treatment for the infection, could result in a hesitancy toward HPV testing and may cause women to question its reliability over cytology [26].

Successful messaging about early detection, plus an understanding of the success of the previous cervical screening program, has led to resistance to change. Women hold so much value to the Pap smear that some would continue to have it regardless of whether it is funded by the government. There was also a suggestion that those who could afford to, would still have more regular screening. However, in the United States, overscreening persists in the under- and uninsured [10].

Resistance to deintensifying screening programs has been demonstrated previously in the United States [10,11] with similar concerns demonstrated in these findings. Although differences exist among breast, cervical, and bowel cancer screening programs, there is likely to be considerable overlap in dealing with concerns for deintensification. For example, concerns women have about increasing the length of interval between screens would likely be a common concern across all 3 screening programs owing to the message about early detection. Equally, with increasing the age of invitation for screening, there will be a focus on *missing* cancers in that age group that is no longer being screened, evident in this study by women concerned about both younger and older women. Introducing a change in test also requires explaining the difference between the old and the new test and the reason for change. Therefore, the recommendations given in this study could be applied to other screening programs, with subtle differences around the physical and psychological impact on individuals recognized.

### Conclusions

Key features of the changes that elicited concern and may apply to other screening programs that undergo deintensification can provide lessons for the future. The most concerning change was regarding the increased screening interval, from 2 to 5 years, with further concern about the increased age of the first invitation to screen. The rationale behind these types of changes in the future needs to be communicated clearly to the public in an effort to increase understanding and alleviate concerns. n addition, communication of the benefits and harms of screening along with resultant overdiagnosis and overtreatment, is necessary to ensure the public are fully informed about screening decisions. We have outlined some recommendations (Table 3) for communicating about deintensifying screening programs, which would help improve the understanding and alleviate concerns.

## Appendix

Multimedia Appendix 1 Content of the petition.

## Abbreviations

HPV: human papillomavirus
NCSP: National Cervical Screening Program
