# Supplementary material for: A Thematic Analysis of Attitudes Toward Changes to Cervical Screening in Australia
Source: JMIR Cancer. 2019 Apr 11;5(1):e12307. doi: 10.2196/12307 (PMC6482401; doi:10.2196/12307)
Supplement: Multimedia Appendix 1 [file cancer_v5i1e12307_app1.pdf]

Appendix 1: Content of the petition

This is a copy of the text which was included in the petition which was opposed to the changes to the Australian National Cervical Screening Program. The final signature count was 71 582.

May 1st Changes to Pap Smears

As of May 1st the following changes will be implemented to women's Pap Smears:

"-women will be invited when they are due to participate via the National Cancer Screening Register
-the time between tests will change from two to five years
-the age at which screening starts will increase from 18 years to 25 years
-women aged 70 to 74 years will be invited to have an exit test.

The new program will commence from 1 May 2017 when the new Cervical Screening Test will become available on the Medicare Benefits Schedule. Until this time, women aged between 18 and 69 years who have ever been sexually active, should continue to have Pap test when due."

The government run website states that:

"Based on new evidence and better technology, the National Cervical Screening Program will change from 1 May 2017 to improve early detection and save more lives."

Prior to May 1 the website advises:

"It is very important that women continue to participate in the current two yearly Pap test program to ensure they are not at risk of developing cervical cancer.

Pap tests have already halved the incidence and mortality from cervical cancer since the introduction of the National Cervical Screening Program in 1991.

Women will be due for the first Cervical Screening Test two years after their last Pap test."

"The new Cervical Screening Test detects human papillomavirus (HPV) infection, which is the first step in developing cervical cancer.

The procedure for collecting the sample for HPV testing is the same as the procedure for having a Pap smear. A Health Care Professional will still take a small sample of cells from the woman's cervix. The sample will be sent to a pathology laboratory for examination.

While the current Pap test can detect abnormal cell changes, the new Cervical Screening Test will detect the HPV infection that can cause the abnormal cell changes, prior to the development of cancer.

Persistent HPV infections can cause abnormal cell changes that may lead to cervical cancer. However, this usually takes a long time, often more than 10 years."

Some of the reasoning behind the changes are:

" -cervical cancer in young women is rare (in both HPV vaccinated and unvaccinated women)
-despite screening women younger than 25 years of age for over 20 years there has been no change to the rates of cervical cancer or rates of death from cervical cancer in this age group
-investigating and treating common cervical abnormalities in young women that would usually resolve by themselves can increase the risk of pregnancy complications later in life
-the HPV vaccination has already been shown to reduce cervical abnormalities among women younger than 25 years of age and, in contrast to screening, is ultimately expected to reduce cervical cancer in this age group."
